# Natural Cyclooxygenase-2 Inhibitors Synergize With Butyrate to Augment Chicken Host Defense Peptide Gene Expression

**DOI:** 10.3389/fimmu.2022.819222

**Published:** 2022-02-22

**Authors:** Qing Yang, Amanda C. Burkardt, Lakshimi T. Sunkara, Kan Xiao, Guolong Zhang

**Affiliations:** ^1^ Department of Animal and Food Sciences, Oklahoma State University, Stillwater, OK, United States; ^2^ Veterinary Diagnostic Center, Clemson University, Clemson, SC, United States; ^3^ Hubei Key Laboratory of Animal Nutrition and Feed Science, Hubei Collaborative Innovation Center for Animal Nutrition and Feed Safety, Wuhan Polytechnic University, Wuhan, China

**Keywords:** cyclooxygenase-2 inhibitors, host defense peptides, antimicrobial peptides, antibiotic alternatives, polyphenols, antimicrobial resistance

## Abstract

Enhancing the synthesis of microbicidal and immunomodulatory host defense peptides (HDP) is a promising host-directed antimicrobial strategy to combat a growing threat of antimicrobial resistance. Here we investigated the effect of several natural cyclooxygenase-2 (COX-2) inhibitors on chicken HDP gene regulation. Our results indicated that phenolic COX-2 inhibitors such as quercetin, resveratrol, epigallocatechin gallate, anacardic acid, and garcinol enhanced HDP gene expression in chicken HTC macrophage cell line and peripheral blood mononuclear cells (PBMCs). Moreover, these natural COX-2 inhibitors showed a strong synergy with butyrate in augmenting the expressions of multiple HDP genes in HTC cells and PBMCs. Additionally, quercetin and butyrate synergistically promoted the expressions of mucin-2 and claudin-1, two major genes involved in barrier function, while suppressing lipopolysaccharide-triggered interleukin-1β expression in HTC macrophages. Mechanistically, we revealed that NF-κB, p38 mitogen-activated protein kinase, and cyclic adenosine monophosphate signaling pathways were all involved in the avian β-defensin 9 gene induction, but histone H4 was not hyperacetylated in response to a combination of butyrate and quercetin. Because of their HDP-inducing, barrier-protective, and antiinflammatory activities, these natural COX-2 inhibitors, when combined with butyrate, may be developed as novel host-directed antimicrobial therapeutics.

## Introduction

The rapid emergence of antibiotic-resistant bacteria has become a major public health concern (1), creating an urgent need for the development of novel antimicrobial therapeutic strategies with a minimum risk to trigger resistance (2). Boosting host innate immunity through induction of endogenous host defense peptide (HDP) synthesis has attracted an increasing attention as a host-directed antiinfective approach (3–5). HDPs, also known as antimicrobial peptides, are a group of small cationic and amphipathic peptides with preferential expression in phagocytes and epithelial cells of the host (6). HDPs consist mainly of the defensin or cathelicidin families in vertebrates. Defensins are categorized by the presence of six cysteine residues, while cathelicidins are comprised of a conserved cathelin domain and a diversified mature peptide sequence (4, 7). A total of 14 defensins (AvBD1–14) and four cathelicidins (CATH1–3 and CATHB1) exist in chickens (7, 8). Chicken HDPs are widely expressed in the respiratory, gastrointestinal, and urogenital tracts as well as in multiple lymphoid cells, and regulated differentially in response to infection and inflammation (7, 8). With antimicrobial, immunomodulatory, and barrier protective activities, HDPs constitute a critically important component of the innate immunity system (6, 9). A range of small-molecule compounds such as short-chain fatty acids, vitamin D_3_, and histone deacetylase (HDAC) inhibitors have been found to be capable of inducing HDP synthesis and some have been explored for disease control and prevention (3–5, 10, 11).

Butyrate, a short-chain fatty acid fermented from dietary carbohydrates by intestinal bacteria, is a well-known HDP inducer in humans and animals (4, 5). Inhibition of HDAC is a major mechanism by which butyrate promotes HDP expression (12), while mitogen-activated protein kinase (MAPK) signaling pathways are also involved in butyrate-mediated HDP induction (13). Butyrate has been shown to confer protection to infections at least in part *via* induction of HDPs (14, 15). Synergic augmentation of HDPs has been observed between butyrate and several other HDP-inducing compounds such as vitamin D_3_ (16), lactose (17, 18), forskolin (19, 20), and wortmannin (21).

Cyclooxygenase (COX) is a key enzyme responsible for the conversion of arachidonic acid to prostanoids (22, 23). COX-1 is a constitutive isoform involved in maintaining barrier integrity, while COX-2 is inducible by inflammatory stimuli and often a target for treating inflammatory diseases. Several natural phenolic compounds such as quercetin, resveratrol, and epigallocatechin gallate (EGCG), anacardic acid, and garcinol are well-known COX-2 inhibitors (22, 23). In addition, a few of these polyphenols such as resveratrol (24), curcumin (25), EGCG (26, 27), and genistein (28) are capable of inducing HDP genes. It is unknown whether these polyphenols induce HDP genes by acting as COX-2 inhibitors. Additionally, a potential HDP-inducing synergy between butyrate and COX-2 inhibitors is yet to be explored.

In the current study, we investigated a potential synergy between butyrate and several natural phenolic COX-2 inhibitors in regulating the expression of various HDP genes in chicken HTC macrophages and peripheral blood mononuclear cells (PBMCs). To confirm these polyphenols synergize with butyrate in HDP induction through COX-2 inhibition, two synthetic COX-2-specific inhibitors namely nimesulide and niflumic acid were also evaluated for their synergy with butyrate in HDP induction in chicken cells. The involvement of histone acetylation as well as the MAPK, nuclear factor-κB (NF-κB), and cyclic adenosine monophosphate (cAMP) signaling pathways in the avian β-defensin 9 (*AvBD9*) gene induction mediated by butyrate and COX-2 inhibitors was also examined.

## Materials And Methods

### Chemicals

Quercetin and garcinol were acquired from Cayman Chemical (Ann Arbor, MI), while sodium butyrate, tributyrin (glyceryl tributyrate), and lipopolysaccharide (LPS) (*Escherichia coli* O55:B5) were obtained from MilliporeSigma (St. Louis, MO). Resveratrol, anacardic acid, EGCG, nimesulide, niflumic acid, SB203580, SP600125, PD98059, PDTC, MG132, and 2’,5’-dideoxyadenosine (DDA) were procured from Santa Cruz Biotechnology (Dallas, TX). Sodium butyrate was dissolved in Roswell Park Memorial Institute (RPMI) 1640 medium (Hyclone, Logan, UT), and all other chemicals were dissolved in dimethyl sulfoxide (DMSO, Santa Cruz Biotechnology). All chemical stock solutions were stored at –20°C or –80°C. An equal volume of RPMI or DMSO was used in all cell stimulation assays as a negative control.

### Culture and Stimulation of Chicken HTC Macrophages

Chicken HTC macrophage cells (29), kindly provided by Dr. Narayan C. Rath at United States Department of Agriculture–Agricultural Research Service, were cultured in complete RPMI 1640 medium containing 10% fetal bovine serum (Atlanta Biologicals, Flowery Branch, GA), 1% streptomycin-penicillin (Lonza, Walkersville, MD) at 1 × 10^6^ cells/well in 6-well tissue culture plates overnight at 37°C and 5% CO_2_ prior to 24-h treatment with different concentrations of natural or synthetic COX-2 inhibitors separately or in combination with 2 mM sodium butyrate or 0.25 mM tributyrin (19, 30, 31), followed by total RNA extraction and expression analyses of chicken HDP genes as described below. HTC cells were also stimulated with butyrate, quercetin, or their combination for 24 h, followed by 3-h stimulation with 10 ng/mL LPS and subsequent expression analyses of the genes involved in barrier function and inflammation.

### Isolation, Culture, and Stimulation of Chicken Peripheral Blood Mononuclear Cells (PBMCs)

Chicken PBMCs were isolated from the peripheral blood of 2- to 4-week-old male Cobb broilers through density gradient centrifugation using Histopaque^®^-1077 (MilliporeSigma) following the manufacturer’s instructions. Briefly, EDTA-anticoagulated blood was overlaid onto an equal volume of Histopaque^®^-1077 and centrifuged at 400 × *g* for 30 min. The interface containing PBMCs was carefully aspirated and if necessary, red blood cells were hypotonically lysed with 0.2% sodium chloride and reconstituted with 1.6% sodium chloride. After three washes in phosphate buffered saline, PBMCs were suspended in complete RPMI 1640 medium containing 10% fetal bovine serum, 1% streptomycin-penicillin, and 20 mM HEPES (Hyclone). After 2-h seeding at 5 × 10^7^ cells/well in 6-well plates, PBMCs were stimulated with various chemicals individually or in combination for 24 h. In the signaling experiments, PBMCs were pre-treated for 1 h with individual specific inhibitors, including 25 µM SB203580 (p38 MAPK inhibitor), 20 µM SP600125 (JNK inhibitor), 50 µM PD98059 (MEK1/2 inhibitor), 40 µM PDTC (NF-κB inhibitor), 20 µM MG132 (NF-κB inhibitor), or 100 or 500 µM DDA (adenylyl cyclase inhibitor) (19, 31), followed by stimulation with 10 µM quercetin and 2 mM butyrate for 24 h.

### RNA Isolation and Reverse Transcriptase-Quantitative PCR (RT-qPCR)

Cells were lysed in RNAzol (Molecular Research Center, Cincinnati, OH) for total RNA isolation according to the manufacturer’s protocol. The quantity and quality of total RNA were determined using Nanodrop (Nanodrop Products, Wilmington, DE). Reverse transcription was performed using QuantiTect Reverse Transcription Kit (Qiagen, Hilden, Germany) following the instructions of the manufacturer. Briefly, genomic DNA was removed from total RNA with gDNA Wipeout Buffer provided in the kit prior to reverse transcription at 42°C for 30 min. The resulting cDNA was then diluted with RNase-free water and used in subsequent qPCR analysis with QuantiTect SYBR Green PCR Kit (Qiagen) according to the manufacturer’s instruction. PCR was performed in 10-µL reactions with initial activation at 95°C for 10 min, followed by 40 cycles at 94°C for 15 s, 55°C for 20 s, and 72°C for 30 s in CFX Real-Time PCR Detection System (Bio-Rad, Hercules, CA). The primer sequences for chicken HDP genes, claudin 1 (*CLDN1*), mucin 2 (*MUC2*), interleukin-1β (*IL-1β*), and glyceraldehyde-3-phosphate dehydrogenase (*GAPDH*) were previously described (14, 18, 20). The specificity of PCR amplification was confirmed using melt curve analysis, and the *CLDN1* and *MUC2* amplicons were further verified through direct Sanger sequencing using gene-specific forward primers. Relative fold changes in gene expressions were calculated using the ΔΔCt method (32) normalized against *GAPDH* as described (14, 18–20).

### Western Blot Analysis

Chicken HTC cells were treated with 2 mM butyrate, 20 μM quercetin, or their combination for 6 or 12 h, followed by lysis in the radioimmunoprecipitation (RIPA) lysis buffer (Santa Cruz Biotechnology). The resulting proteins were quantified using the Bradford Assay (Bio-Rad), followed by loading of 20 µg proteins from each sample in 12.5% SDS-PAGE gels and transferring to Immobilon-P^®^ polyvinylidene difluoride membranes (MilliporeSigma). After overnight blocking in 5% dry skim milk in TTBS (0.05% Tween 20, 20 mM Tris-HCl, 150 mM NaCl, pH 7.5) at 4°C, the membranes were incubated with a primary rabbit antibody against acetyl-histone H4 (Cell Signaling, Danvers, MA; diluted 1:500) or a rabbit antibody against β-Actin (MilliporeSigma; diluted 1:1000) in the blocking buffer for 1 h at room temperature. After three washes in TTBS, the membranes were incubated with an alkaline phosphatase-conjugated goat anti-rabbit IgG antibody (MilliporeSigma; diluted 1:2,000) for 45 min at room temperature, followed by visualization using enhanced chemiluminescence (ThermoFisher Scientific). The band intensity of acetyl-histone H4 was quantified as the area under the curve using ImageJ (https://imagej.nih.gov/ij/) and further normalized against the band intensity of β-Actin for each sample.

### Statistical Analysis

Statistical analysis and data visualization were performed using GraphPad Prism (GraphPad Software, La Jolla, CA). The results were expressed as means ± standard error of the mean (SEM) from 2–3 independent experiments. One-way ANOVA and *post hoc* Tukey’s test were applied to determine statistical significance. The results were considered statistically significant if *P* < 0.05.

## Results

### Concentration- and Time-Dependent Induction of *AvBD9* Gene Expression in Response to Quercetin

**Figure 1:**
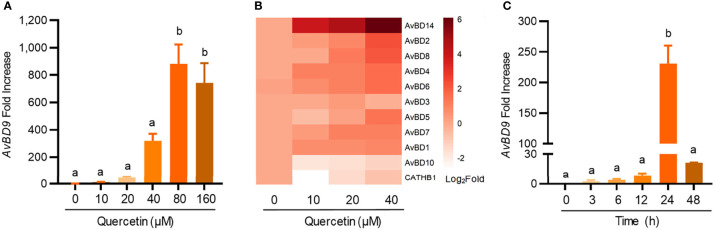
Dose- and time-dependent induction of chicken host defense peptide (HDP) gene expressions by quercetin. Chicken HTC macrophage cells were treated in duplicate with indicated concentrations of quercetin for 24 h, followed by RT-qPCR analysis of avian β-defensin 9 (*AvBD9*) **(A)** and other HDP genes **(B)**. **(C)** HTC cells were also treated with 40 µM quercetin for indicated times, followed by *AvBD9* expression analysis. Results are expressed as means ± SEM of three independent experiments. The bars not sharing common superscript letters are significantly different (*P* < 0.05) as determined by one-way ANOVA and *post hoc* Tukey’s test.

To study whether chicken HDP genes are regulated by quercetin, both dose-response and time-course experiments were conducted with quercetin in chicken HTC macrophage cells. *AvBD9*, the most inducible chicken HDP genes in response to a variety of small-molecule compounds (14, 18, 31), was gradually augmented in response to increasing concentrations of quercetin, reaching a peak of 881-fold induction at 80 μM (**Figure 1A**). In addition, almost all HDP genes that are expressed in HTC cells were induced by quercetin, although the magnitude of induction varied greatly among different genes (**Figure 1B**). For example, *AvBD14* was readily induced, while *AvBD10* showed minimum alterations. An obvious time-dependent induction of *AvBD9* expression was also observed in HTC cells in response to 40 μM quercetin, peaking at 24 h (**Figure 1C**).

### Synergistic Induction of HDP Genes by Quercetin and Butyrate

**Figure 2:**
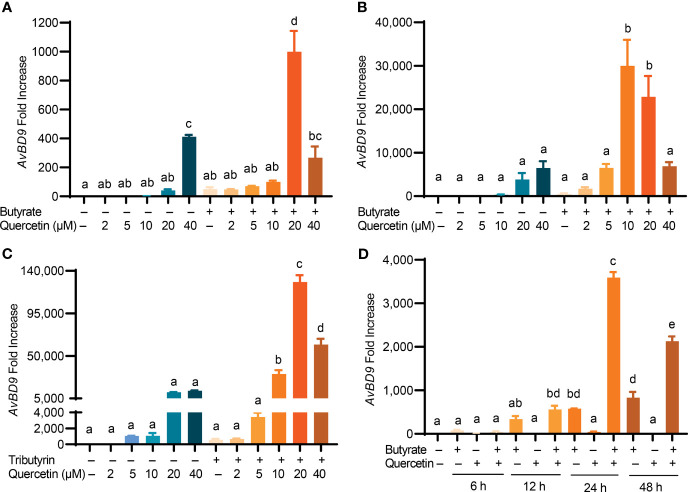
Synergistic induction of *AvBD9* gene expression by a combination of quercetin and butyrate or tributyrin. **(A)** Chicken HTC macrophages or **(B)** peripheral blood mononuclear cells (PBMCs) were treated in duplicate with indicated concentrations of quercetin with or without 2 mM butyrate for 24 h. **(C)** Chicken PBMCs were stimulated with indicated concentrations of quercetin with or without 0.25 mM tributyrin for 24 h. **(D)** Chicken PBMCs were treated in duplicate with 2 mM butyrate and 10 μM quercetin individually or in combination for indicated times. The expression levels of the *AvBD9* gene were analyzed by RT-qPCR. Results are presented as means ± SEM of 2–3 independent experiments. The bars not sharing common superscript letters are significantly different (*P* < 0.05) as determined by one-way ANOVA and *post hoc* Tukey’s test.

**Figure 3:**
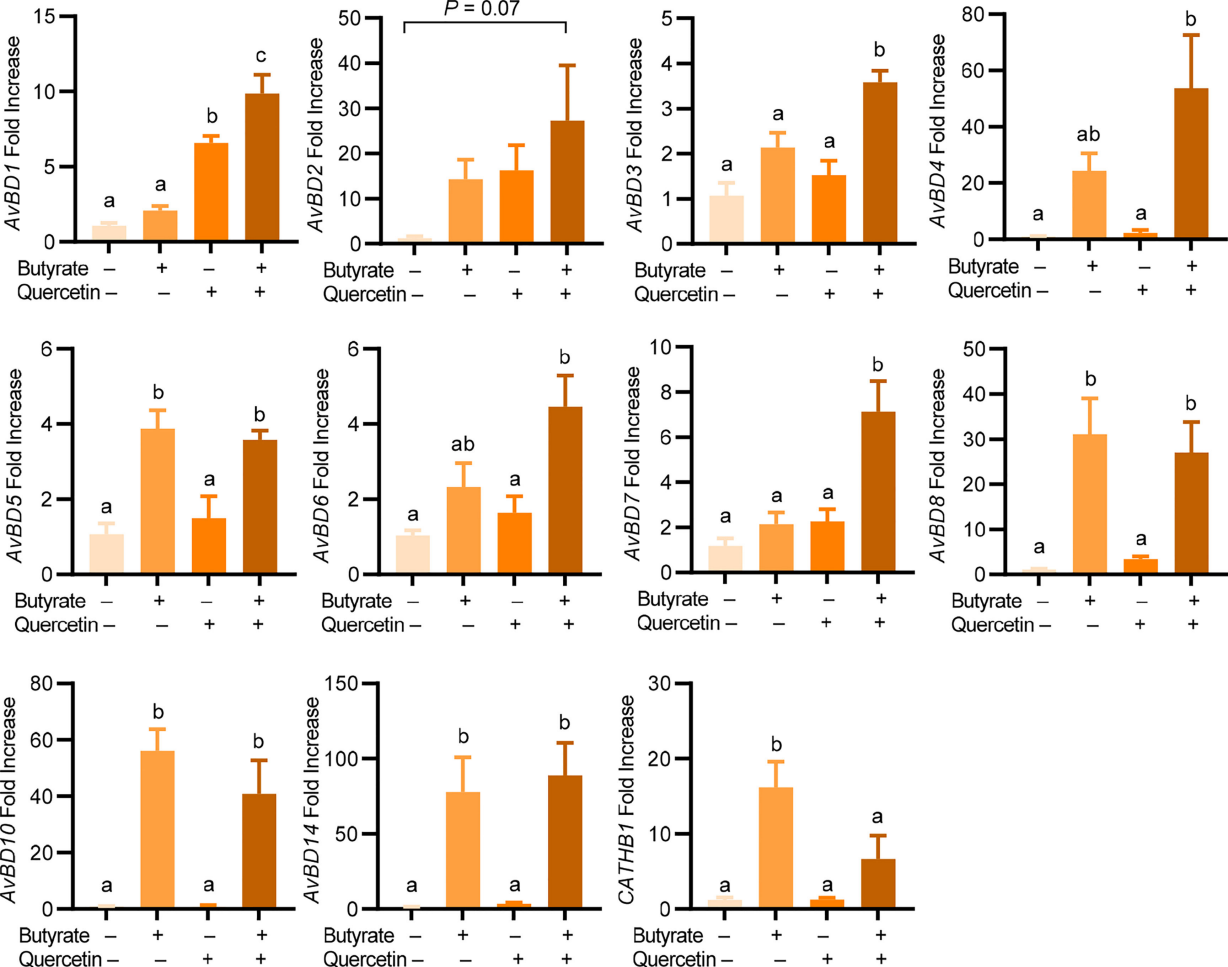
Induction of chicken HDP genes by quercetin and butyrate. Chicken HTC macrophage cells were treated in duplicate with 20 μM quercetin and 2 mM sodium butyrate separately or in combination for 24 h, followed by RT-qPCR analysis of chicken HDP gene expression. Results are presented as means ± SEM of two independent experiments. The bars not sharing common superscript letters are significantly different (*P* < 0.05) as determined by one-way ANOVA and *post hoc* Tukey’s test.

To explore a possible synergy in HDP induction between quercetin and butyrate, chicken HTC cells were treated with different concentrations of quercetin in the presence or absence of 2 mM sodium butyrate for 24 h. A clear synergy was observed with a peak 1000-fold induction when 2 mM butyrate was combined with 20 µM quercetin, while they separately gave a 50- and 41-fold *AvBD9* induction, respectively (**Figure 2A**). To confirm the quercetin/butyrate synergy in HDP induction in a different cell type, chicken PBMCs were treated with butyrate and quercetin individually or in combination. Similar to HTC macrophages, quercetin alone elevated *AvBD9* transcription in a dose-dependent fashion and more importantly, synergized dramatically with butyrate (**Figure 2B**). It is noteworthy that the synergism was diminished when butyrate was combined with higher concentrations of quercetin in both HTC cells (**Figure 2A**) and PBMCs (**Figure 2B**). Quercetin also synergized strongly with tributyrin, a triglyceride analog of butyrate (33), in improving *AvBD9* expression in chicken PBMCs (**Figure 2C**). To study the kinetics of the butyrate/quercetin synergy in HDP induction, a time-course experiment was conducted using quercetin and butyrate separately or in combination. The synergy became more pronounced as the treatment time was gradually increased, with a 24-h treatment giving the strongest synergy in inducing *AvBD9*, while a longer 48-h stimulation caused a diminished synergy in HTC cells (**Figure 2D**). Besides *AvBD9*, butyrate and quercetin also synergistically increased *AvBD1*, *AvBD2*, *AvBD3*, *AvBD4*, *AvBD6*, and *AvBD7*, but not *AvBD5*, *AvBD8*, *AvBD10*, *AvBD14*, or *CATHB1*, in HTC macrophages (**Figure 3**). Notably, varying magnitudes of induction were observed for different HDP genes (**Figure 3**), indicating a gene-specific effect.

### Modulation of Barrier Function and Inflammatory Cytokine Gene Expression by Butyrate and Quercetin

**Figure 4:**
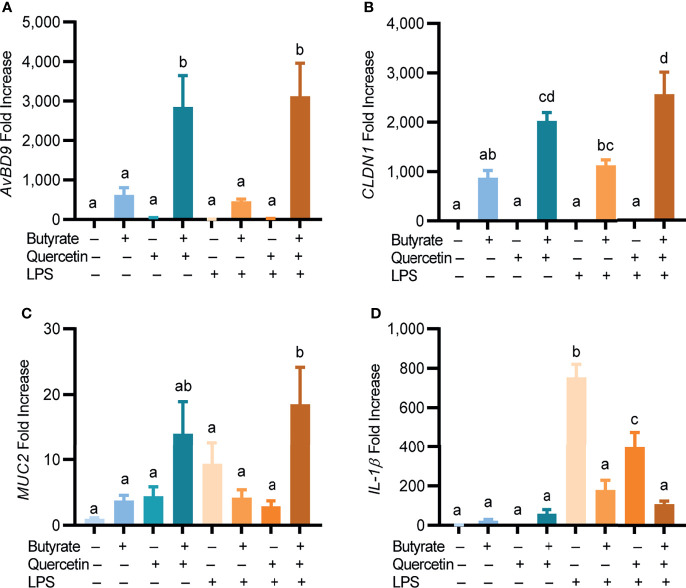
Regulation of HDP, barrier function, and inflammatory cytokine gene expressions by quercetin and butyrate. Chicken HTC macrophage cells were treated in duplicate with 20 μM quercetin and 2 mM sodium butyrate separately or in combination for 24 h, followed by stimulation with 10 ng/mL lipopolysaccharides (LPS) for another 3 h. Gene expressions of *AvBD9* **(A)**, claudin 1 (*CLDN1*) **(B)**, mucin 2 (*MUC2*) **(C)**, and interleukin 1β (*IL-1β*) **(D)** were analyzed by RT-qPCR. Results are presented as means ± SEM of two independent experiments. The bars not sharing common superscript letters are significantly different (*P* < 0.05) as determined by one-way ANOVA and *post hoc* Tukey’s test.

Butyrate and quercetin are known to enhance barrier function by inducing mucin and tight junction protein expressions while suppressing inflammation (34, 35). CLDN1 is critically involved in tight junction assembly (35), while MUC2 is the most abundant component of the intestinal mucus layer (36). To investigate a potential synergy between butyrate and quercetin in regulating barrier function and inflammation, chicken HTC macrophage cells were treated for 24 h with butyrate and quercetin separately or in combination prior to a 3-h stimulation with LPS. A synergistic induction of *AvBD9* by butyrate and quercetin was obviously maintained following LPS stimulation (**Figure 4A**). While 2 mM butyrate induced *CLDN1* gene expression by 875-fold and 20 μM quercetin showed a minimum effect, the butyrate/quercetin combination elicited a synergistic 2,024-fold increase in *CLDN1* expression, which was unaffected by LPS (**Figure 4B**). Moreover, butyrate or quercetin improved the *MUC2* mRNA expression individually, and the combination synergistically enhanced *MUC2* expression even in the presence of LPS (*P* < 0.05) (**Figure 4C**). Additionally, butyrate, quercetin, or the combination had no significant impact on *IL-1β* expression. As expected, butyrate or quercetin suppressed LPS-induced *IL-1β* expression in HTC cells (*P* < 0.05). Desirably, the butyrate/quercetin combination had the strongest effect in reducing LPS-triggered *IL-1β* expression (**Figure 4D**). Collectively, quercetin is synergistic with butyrate in boosting the expressions of *AvBD9*, *CLDN1*, and *MUC2*, while suppressing *IL-1β* during LPS-triggered stimulation. It is noted that LPS had no effect on *AvBD9* expression, but triggered a 754-fold induction of *IL-1β* gene expression (**Figures 4A, D**).

### Synergistic Augmentation of *AvBD9* Expression by Butyrate and Other COX-2 Inhibitors

**Figure 5:**
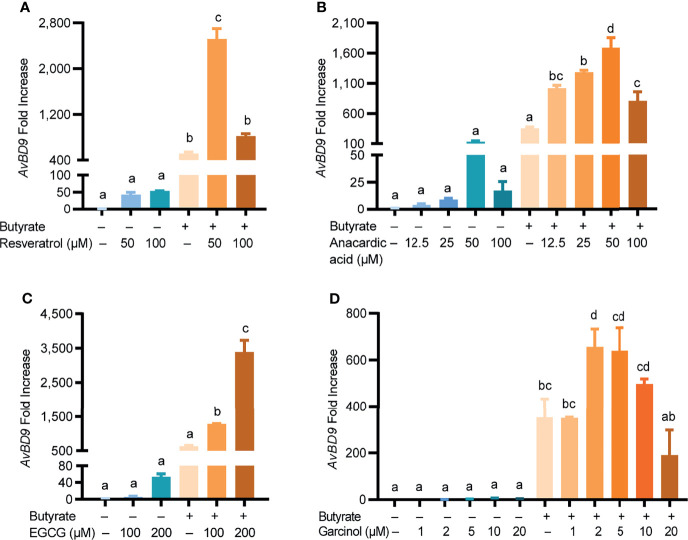
Induction of *AvBD9* by natural COX-2 inhibitors and butyrate. Chicken PBMCs were treated in duplicate with indicated concentrations of resveratrol **(A)**, anacardic acid **(B)**, epigallocatechin gallate (EGCG) **(C)**, or garcinol **(D)**, in the presence or absence of 2 mM butyrate for 24 h, followed by *AvBD9* expression analysis. Results are presented as means ± SEM of 2–3 independent experiments. The bars not sharing common superscript letters are significantly different (*P* < 0.05) as determined by one-way ANOVA and *post hoc* Tukey’s test.

To further evaluate the synergy between butyrate and other COX-2 inhibitors in chicken HDP gene induction, four additional natural COX-2 inhibitors including resveratrol, anacardic acid, EGCG, and garcinol were tested in chicken PBMCs with or without butyrate. Similar to quercetin, each compound dose-dependently elevated *AvBD9* expression and also synergized markedly with butyrate in *AvBD9* gene expression (**Figure 5**). For example, 50 µM resveratrol and 2 mM butyrate induced *AvBD9* expression by 43- and 511-fold, respectively, while the combination augmented *AvBD9* expression by 2,519-fold (**Figure 5A**). Similarly, anacardic acid (**Figure 5B**), EGCG (**Figure 5C**), and garcinol (**Figure 5D**) also demonstrated a strong synergy with butyrate in *AvBD9* mRNA induction, although garcinol was relatively weak in its ability to synergize with butyrate.

**Figure 6:**
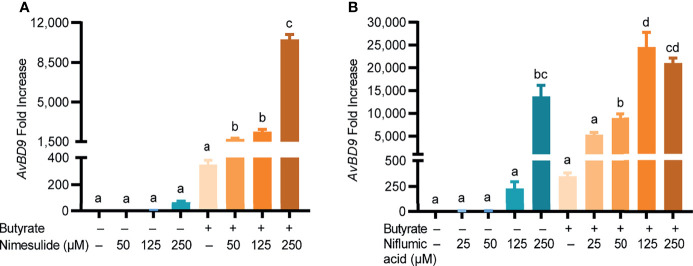
Synergistic induction of *AvBD9* gene expression by synthetic COX-2-specific inhibitors and butyrate. Chicken PBMCs were treated in duplicate with indicated concentrations of nimesulide **(A)** or niflumic acid **(B)** with or without 2 mM butyrate for 24 h, followed by RT-qPCR analysis of *AvBD9* gene expression. Results are presented as means ± SEM of two independent experiments. The bars not sharing common superscript letters are significantly different (*P* < 0.05) as determined by one-way ANOVA and *post hoc* Tukey’s test.

In addition to acting as a COX-2 inhibitor, quercetin, resveratrol, anacardic acid, EGCG, and garcinol also exert a range of other effects on host cells (37, 38). To confirm COX-2-specific inhibitors can also synergize with butyrate in HDP induction, two highly selective COX-2 inhibitors namely nimesulide and niflumic acid (39, 40) were separately applied to chicken PBMCs with or without butyrate. Similar to their natural counterparts, both nimesulide and niflumic acid increased *AvBD9* gene expression in a dose-dependent manner, further with a marked synergy with butyrate (**Figure 6**). For example, 250 µM nimesulide and 2 mM butyrate together enhanced *AvBD9* gene expression by 10,524-fold, while they individually gave 69- and 349-fold induction, respectively (**Figure 6A**). A robust synergy also occurred between niflumic acid and butyrate (**Figure 6B**). Taken together, inhibition of the COX-2 pathway upregulates *AvBD9* gene expression and further synergizes with butyrate to promote *AvBD9* transcription.

### Involvement of Histone Acetylation and MAPK, NF-κB, and cAMP Signaling in *AvBD9* Induction by Butyrate and Quercetin

**Figure 7:**
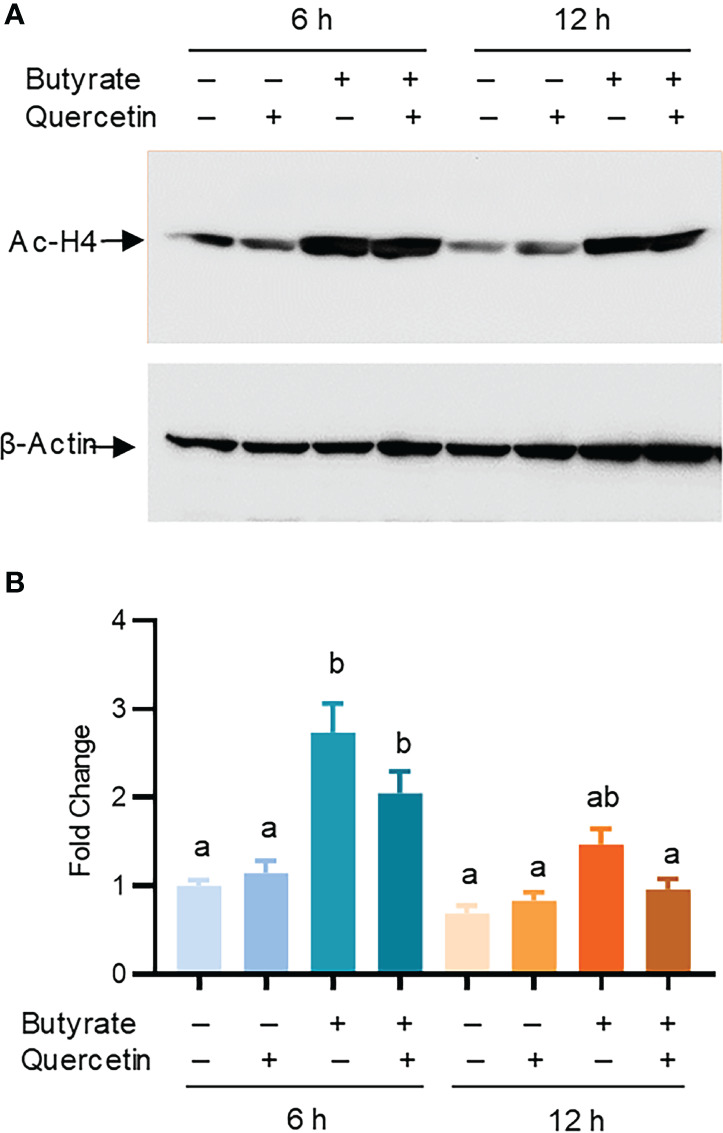
Role of histone acetylation in *AvBD9* induction by butyrate and quercetin. Chicken HTC cells were treated with 2 mM sodium butyrate or 20 μM quercetin individually or in combination for 6 or 12 h, followed by western blot analysis of acetyl-histone H4 (Ac-H4) and β-actin. **(A)** A representative of two independent blots. **(B)** Fold changes in the acetylation of histone H4 relative to unstimulated cells. Results are presented as means ± SEM of two independent experiments normalized against β-actin. The bars not sharing common superscript letters are significantly different (*P* < 0.05) as determined by one-way ANOVA and *post hoc* Tukey’s test.

Butyrate is a well-known HDAC inhibitor to maintain the hyper-acetylation status of histones to facilitate chromatin relaxation and subsequent gene expression (41). To investigate whether histone acetylation is involved in butyrate/quercetin-mediated synergy in *AvBD9* induction, chicken HTC cells were treated with butyrate and quercetin separately or in combination for 6 and 12 h, followed by examination of histone 4 (H4) acetylation using Western blotting. Butyrate, but not quercetin, significantly increased H4 acetylation at 6 h and the acetylation was diminished at 12 h (**Figures 7A, B**). Relative to butyrate, a combination of butyrate and quercetin showed no hyper-acetylation at either time point (**Figures 7A, B**), suggesting that the HDP-inducing synergy between butyrate and quercetin is not caused by hyper-acetylation of histones in the HDP gene promoters.

**Figure 8:**
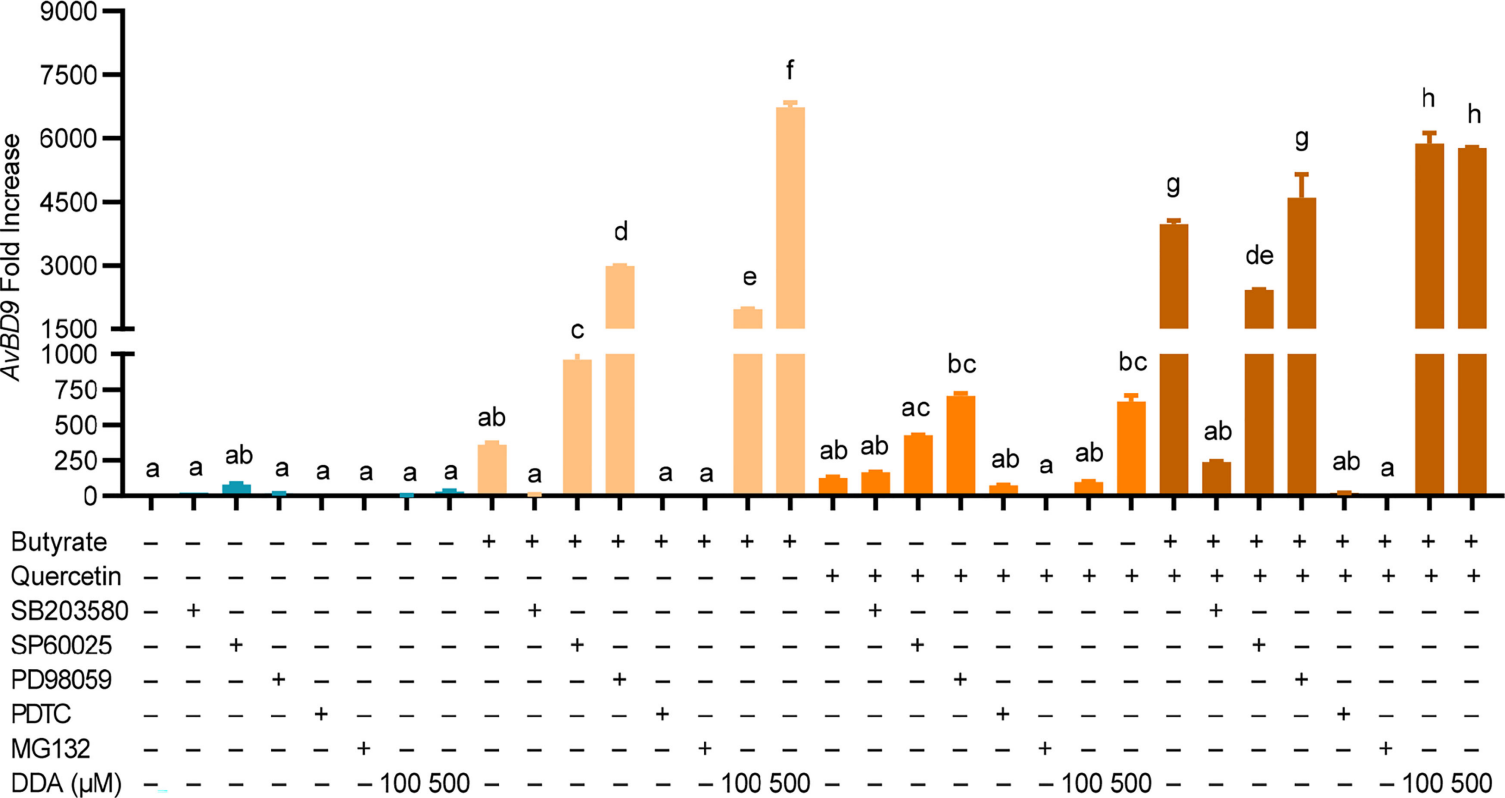
Role of MAPK, NF-κB, and cAMP signaling pathways in *AvBD9* induction mediated by quercetin and butyrate. Chicken PBMCs were pretreated for 1 h with or without 25 µM SB203580 (p38 MAPK inhibitor), 20 µM SP60025 (JNK inhibitor), 50 µM PD98059 (MEK1/2 inhibitor), 40 µM PDTC (NF-κB inhibitor), 20 µM MG132 (NF-κB inhibitor), or 100 or 500 µM 2’,5’-dideoxyadenosine (DDA) (cAMP inhibitor), followed by stimulation with 10 µM quercetin with or without 2 mM sodium butyrate for another 24 h. RT-qPCR was performed to determine *AvBD9* mRNA expression. Results are presented as means ± SEM of two independent experiments. The bars not sharing common superscript letters are significantly different (*P* < 0.05) as determined by one-way ANOVA and *post hoc* Tukey’s test.

To examine the role of MAPK, NF-κB, and cAMP signaling in *AvBD9* induction, chicken PBMCs were treated with quercetin and butyrate in the presence or absence of specific inhibitors for three major MAPK pathways as well as NF-κB, and cAMP pathways. To our surprise, inhibition of three MAPK pathways (with SB203580, SP60025, or PD98059) modestly activated the basal expression of the *AvBD9* gene (**Figure 8**), suggesting that blocking MAPK signaling is beneficial for the constitutive expression of *AvBD9*. However, inhibiting the p38 MAPK pathway with SB203580 gave a substantial reduction in butyrate-induced *AvBD9* expression, while blocking the JNK and MEK1/2 MAPK pathways with SP60025 and PD98059, respectively, potentiated *AvBD9* expression (**Figure 8**), implying that p38, but not JNK or MEK1/2 MAPK, is required for butyrate-mediated *AvBD9* induction. However, p38 MAPK appeared to have no effect on quercetin-induced *AvBD9* expression, while suppressing the JNK and MEK1/2 pathways increased *AvBD9* expression. Blocking p38 MAPK substantially reduced *AvBD9* gene expression in chicken PBMCs in response to a combination of butyrate and quercetin, while inhibiting the JNK and MEK-ERK pathways had a minimum effect on *AvBD9* expression (**Figure 8**). These results suggest a differential involvement of three canonical MAPK signaling pathways in *AvBD9* induction mediated by butyrate, quercetin, or the combination.

Blocking NF-κB with PDTC or MG132 had no impact on the basal expression of *AvBD9*, but caused nearly a complete abolishment of *AvBD9* expression induced by butyrate, quercetin, or the combination (**Figure 8**), indicating a critical role of NF-κB in *AvBD9* gene expression. Inhibition of the cAMP pathway with DDA resulted in a modest *AvBD9* induction in PBMCs under the basal condition; however, DDA dose-dependently increased *AvBD9* expression induced by butyrate or quercetin and further potentiated *AvBD9* expression in response to the butyrate/quercetin combination (**Figure 8**), implying that inhibiting cAMP signaling is beneficial for *AvBD9* induction mediated by butyrate, quercetin, or the combination.

## Discussion

Enhancing HDP synthesis is a promising host-directed approach to antimicrobial therapy (3–5, 11). This study revealed a potent HDP-inducing activity of several natural phenolic compounds with COX-2 inhibitory activity. We further reported a strong synergy between butyrate and these natural COX-2 inhibitors in promoting HDP expression and barrier function while suppressing LPS-mediated inflammatory response in chicken cells. These multifaceted beneficial effects of polyphenols and butyrate make them attractive candidates for further evaluation of their efficacy in disease control and prevention in chickens and possibly other animals.

COX-2 is a membrane-bound enzyme responsible for the production of prostanoids including prostaglandins and thromboxanes from arachidonic acid during inflammation (22, 23). As a result, a variety of cell type-specific prostaglandins are synthesized and in turn lead to inflammation and pain (22, 23). Specific inhibition of COX-2 can be achieved by using nonsteroidal anti-inflammatory drugs (NSAIDs) (22, 23). Many polyphenols have COX-2 inhibitory activity (42). For example, quercetin is capable of suppressing COX-2 gene expression and prostaglandin E2 (PGE2) production (43). Recently, COX-2 signaling was linked to HDP expression. Activation of COX-2 downregulates human β-defensin 1 (*DEFB1*) mRNA expression in intestinal epithelial cells (44), and PGE2 is known to suppress human β-defensin 2 (*DEFB4*) expression in gingival epithelial cells (45) and human cathelicidin antimicrobial peptide (*CAMP*) gene in macrophages (46). Conversely, inhibiting COX-2 increases *DEFB4* expression in human gingival epithelial cells (45). Several polyphenols such as resveratrol and EGCG have also been shown to induce HDP gene expression (24, 26, 27). However, it remains unknown whether these polyphenols induce HDP expression by acting as COX-2 inhibitors.

In the current study, several structurally unrelated polyphenols such as quercetin, anacardic acid, and garcinol were tested in parallel with resveratrol and EGCG. All tested polyphenols possess HDP-inducing activity and further synergize with butyrate in HDP induction. We also confirmed two highly selective COX-2 inhibitors including nimesulide and niflumic acid (39, 47) are also capable of inducing HDP expression showing a synergistic activity with butyrate in promoting HDP gene expression, similar to those polyphenols. These results suggest that polyphenols enhance HDP expression at least in part through inhibition of COX-2 activation, perhaps by suppressing PGE2 synthesis, which is known to downregulate HDP gene expression (45, 46).

Histone acetylation plays an important role in modulating HDP gene expression and many compounds with HDAC inhibitory activity have been found to induce HDP expression (5). It is unsurprising to see butyrate, a well-known HDAC inhibitor, to cause acetylation of histone H4 in chicken HTC cells in this study. Although many polyphenols such as resveratrol, quercetin, and EGCG have epigenetic functions by modifying DNA methylation and histone methylation and acetylation (48), quercetin fails to obviously induce histone acetylation showing no cooperative activity with butyrate in hyper-acetylation of histones, suggesting that its synergy with butyrate in HDP induction is unrelated to histone acetylation.

However, we found that blocking the p38 MAPK signaling pathway substantially abolishes *AvDB9* gene induction in PBMCs mediated by butyrate or the butyrate/quercetin combination, but not quercetin alone; however, inhibiting the MEK1/2 or JNK pathway instead potentiates *AvDB9* gene expression in response to butyrate, quercetin, the combination, or even in the basal quiescent state. Differential involvement of the three canonical MAPK pathways in HDP induction in PBMCs is consistent with earlier observations that three MAPK pathways mediate HDP induction in compound- and cell type-specific manners (11). For example, inhibition of p38 and JNK pathways decreases *AvBD9* expression, while suppressing MEK1/2 increases *AvBD9* expression in response to butyrate or/and forskolin in chicken HD11 macrophage cells (19). On the other hand, resveratrol induces human *CAMP* gene expression through p38 MAPK, while blocking the MEK1/2 pathway further enhances resveratrol-induced *CAMP* expression in human keratinocytes (24).

Phosphorylation of CCAAT-enhancer-binding protein α (C/EBPα) by p38 MAPK is believed to be responsible for resveratrol-mediated *CAMP* expression (24). Whether C/EBPα is also involved in HDP induction by other polyphenols is currently unknown. Given no influence of p38 MAPK on quercetin-mediated *AvBD9* induction, it is likely that different polyphenols induce HDP gene expression differently, but it is also possible that a gene- or cell type-specific HDP regulation pattern exists. One possible reason for HDP induction in response to the MEK1/2 blockage could be due to reduced phosphorylation of cAMP response element-binding protein (CREB), which is a major target of the MEK1/2 pathway (49, 50). Reduced CREB phosphorylation in turn suppresses the synthesis of inducible cAMP early repressor (ICER), which could otherwise compete with CREB for the cAMP response element (CRE) on an HDP gene promoter resulting in reduced gene transcription (51, 52).

NF-κB is also required for *AvBD9* induction mediated by butyrate, quercetin, or the combination, which is in agreement with earlier observations on the involvement of NF-κB in human *CAMP* induction by resveratrol and genistein, where NF-κB is believed to activate p38 MAPK for phosphorylation of C/EBPα and activation of *CAMP* gene transcription (24, 28). It is noted that NF-κB is critical for the induction of the COX-2 gene, and polyphenols block the COX-2 activity and PGE2 synthesis by suppressing NF-κB action (53). Reduced PGE2 production thus leads to increased HDP gene expression (45, 46).

Similar to the MEK1/2 MAPK pathway, inhibition of the cAMP signaling pathway appears to be beneficial for butyrate- and quercetin-mediated *AvBD9* gene induction in chicken PBMCs. Consistently, activation of cAMP signaling suppresses the expression of human *CAMP* and *DEFB1* genes in colonic epithelial cells (44). However, forskolin, an adenylyl cyclase agonist and activator of the cAMP-protein kinase A (PKA) signaling (54), has been found to be a weak inducer of *AvBD9* and even synergize with butyrate in *AvBD9* induction in chicken HD11 macrophages (18–20). The discrepancy is likely due to a difference in the cell type used in different studies, but it is also possible because of a difference in the strength and duration of the cAMP signaling being activated. Forskolin is beneficial to activate the cAMP-PKA pathway to phosphorylate CREB for enhanced HDP synthesis; however, excessive and prolonged CREB activation may sustain ICER synthesis, creating a negative feedback to turn off HDP synthesis (20, 51). Therefore, it is beneficial to fine-tune the amount and duration of CREB synthesis and phosphorylation to maintain a positive ratio of phosphorylated CREB over phosphorylated ICER and increased HDP gene expression. In fact, diminished HDP induction occurs with high concentrations of butyrate or forskolin or prolonged treatment (14, 19, 55), which is associated with increased ICER transcription (20, 51). Similarly, we also observed a diminished synergic *AvBD9* induction in response to a combination of butyrate and high concentrations of natural COX-2 inhibitors in this study. ICER is likely involved in the reduced synergism, but an experimental verification is warranted.

Although HDPs are an important part of the innate immunity, not all HDPs are increasingly transcribed and produced in response to infection or injury. In chicken HTC macrophages, a 3-h LPS treatment triggered a massive *IL-1β* induction, but *AvBD9* gene expression was not altered, which is consistent with earlier reports that *AvBD9* was unaffected in the vagina or Sertoli cells of chickens for up to 24 h following LPS treatment (56, 57), although another report appeared to suggest that *AvBD9* is upregulated by LPS in chicken bone marrow-derived cells (58). The discrepancy is currently unknown, perhaps it is cell type-dependent.

In the current study, we believe that COX-2 inhibition is at least partially responsible for polyphenol-mediated HDP synthesis; however, we cannot rule out relative contributions of other biological activities of individual polyphenols to HDP induction. Most polyphenols have antioxidative, antiinflammatory, and antiproliferative activities by modulating a multitude of biological processes. Besides COX-2 inhibition, NF-κB, MAPK, and signal transducer and activator of transcription proteins (STATs) are all commonly suppressed by polyphenols (42). Additionally, many polyphenols have been shown to influence DNA and histone methylation beyond histone acetylation (48). Therefore, it will be important to dissect the relative contributions of each of those individual activities of polyphenols to HDP induction. It is likely different polyphenols induce HDP expression through different mechanisms beyond COX-2 inhibition. It will be important to expand the testing of additional structurally and functionally distinct polyphenols as well as other highly specific COX-2 inhibitors such as aspirin, ibuprofen, and coxibs (23) for their HDP-inducing activity. Additionally, it is critical to examine whether the HDP-inducing synergy between butyrate and COX-2 inhibitors similarly occurs in other animal species or humans.

## Conclusions

We have revealed that several structurally different polyphenols are potent HDP inducers likely by acting as COX-2 inhibitors. Furthermore, these natural COX-2 inhibitors are synergistic with butyrate in inducing HDP and barrier function gene expression without eliciting inflammation, suggesting their potential for further exploration as a novel host-directed approach to antimicrobial therapy in chickens and possibly other animals, although additional *in vivo* studies are warranted.

## Data Availability Statement

The original contributions presented in the study are included in the article. Further inquiries can be directed to the corresponding author.

## Author Contributions

QY, AB, LS, and KX conducted the experiments. QY, AB, and LS performed data analysis. QY and AB drafted the manuscript. GZ conceived the study and revised the manuscript. All authors contributed to the article and approved the submitted version.

## Funding

This research was funded by the USDA National Institute of Food and Agriculture (grant no. 2018-68003-27462 and 2020-67016-31619), Oklahoma Center for the Advancement of Science and Technology (grant no. AR19-027), the Ralph F. and Leila W. Boulware Endowment Fund, and Oklahoma Agricultural Experiment Station Project H-3112.

## Conflict of Interest

The authors declare that the research was conducted in the absence of any commercial or financial relationships that could be construed as a potential conflict of interest.

## Publisher’s Note

All claims expressed in this article are solely those of the authors and do not necessarily represent those of their affiliated organizations, or those of the publisher, the editors and the reviewers. Any product that may be evaluated in this article, or claim that may be made by its manufacturer, is not guaranteed or endorsed by the publisher.

## Abbreviations

AvBD9, avian β-defensin 9; C/EBPα, CCAAT-enhancer-binding protein α; CAMP, cathelicidin antimicrobial peptide; cAMP, cyclic adenosine monophosphate; CLDN1, claudin 1; COX-2, cyclooxygenase-2; CRE, cAMP response element; CREB, cAMP response element-binding protein; DDA, 2’,5’-dideoxyadenosine; DEFB1, human β-defensin 1; DEFB4, human β-defensin 2; EGCG, epigallocatechin gallate; GAPDH, glyceraldehyde-3-phosphate dehydrogenase; HDAC, histone deacetylase; HDPs, host defense peptides; ICER, inducible cAMP early repressor; IL-1β, interleukin-1β; LPS, lipopolysaccharide; MAPK, mitogen-activated protein kinase; MUC2, mucin 2; NF-κB, nuclear factor-κB; NSAIDs, nonsteroidal anti-inflammatory drugs; PBMCs, peripheral blood mononuclear cells; PGE2, prostaglandin E2; PKA, protein kinase A; RIPA, radioimmunoprecipitation; RPMI, Roswell Park Memorial Institute; RT-qPCR, reverse transcriptase-quantitative PCR; SEM, standard error of the mean; STATs, signal transducer and activator of transcription proteins.
